# Stereotactic Body Radiotherapy for Small Lung Tumors in the University of Tokyo Hospital

**DOI:** 10.1155/2014/136513

**Published:** 2014-07-07

**Authors:** Hideomi Yamashita, Wataru Takahashi, Akihiro Haga, Satoshi Kida, Naoya Saotome, Keiichi Nakagawa

**Affiliations:** Department of Radiology, The University of Tokyo Hospital, 7-3-1 Hongo, Bunkyo-ku, Tokyo 113-8655, Japan

## Abstract

Our work on stereotactic body radiation therapy (SBRT) for primary and metastatic lung tumors will be described. The eligibility criteria for SBRT, our previous SBRT method, the definition of target volume, heterogeneity correction, the position adjustment using four-dimensional cone-beam computed tomography (4D CBCT) immediately before SBRT, volumetric modulated arc therapy (VMAT) method for SBRT, verifying of tumor position within internal target volume (ITV) using in-treatment 4D-CBCT during VMAT-SBRT, shortening of treatment time using flattening-filter-free (FFF) techniques, delivery of 4D dose calculation for lung-VMAT patients using in-treatment CBCT and LINAC log data with agility multileaf collimator, and SBRT method for centrally located lung tumors in our institution will be shown. In our institution, these efforts have been made with the goal of raising the local control rate and decreasing adverse effects after SBRT.

## 1. Introduction

Stereotactic body radiation therapy (SBRT) has been widely used as a safe and effective treatment method for primary or metastatic lung tumors [1]. Favorable initial clinical results and local control rates around 90% have been reported [2–8].

## 2. Materials and Methods

Since March 2003, SBRT has been employed for approximately 230 body trunk tumors using a simple body cast system at the University of Tokyo Hospital. From October 2010, single-arc volumetric modulated arc therapy- (VMAT-) SBRT of coplanar beam using an Elekta-synergy system has been performed. Before that, static noncoplanar 8–11 multiportal beams had been used in SBRT. In this review, our studies on SBRT of the lung will be reviewed.

## 3. Results and Discussion

### 3.1. Eligibility Criteria in Our Institution

In our institution, the eligibility criteria of lung SRT were (1) solitary or double lung tumors; (2) tumor diameter <40 mm; (3) no evidence of regional lymph node metastasis; and (4) Karnofsky performance status scale not less than 80%.

According to the protocol of the Japan Clinical Oncology Group (JCOG) 0403 study [11, 12], the absolute contraindication to SBRT was pregnancy. Other contraindications included (a) a history of irradiation to the concerned site, (b) severe interstitial pneumonitis or pulmonary fibrosis, (c) severe diabetes or connective tissue disease, and (d) common use of steroids. In our institution, these conditions have been followed. However, these complications preclude other treatment methods in some cases since radiation therapy becomes the only available treatment.

In our institution, patients have been prescreened for serum Krebs von den Lungen-6 (KL-6) and serum surfactant protein-D (SP-D) as biomarkers of severe radiation pneumonitis (RP) [13]. Patients presenting with an interstitial pneumonitis (IP) shadow in computed tomography (CT) and a high value (over 500 U/mL) of serum KL-6 before SBRT were excluded, since radiation pneumonitis will occur at a high rate [13].

### 3.2. Previous SRT Method in Our Institution

The axial CT images were transferred to a 3-dimensional RT treatment-planning machine (Pinnacle^3^, New Version 7.4i, Philips). Treatment planning was performed using the 3D RTP machine. The target reference point dose was defined at the isocenter of the beam. The collapsed cone convolution (CCC) method was used as the dose calculation, in which the range of Compton electrons was better taken into account. The planned dose at the isocenter was 48 Gy in four fractions [biological effective dose (*α*/*β* = 10 Gy) (BED_10_) = 105.6 Gy] for peripheral lesions or 56 Gy in seven fractions (BED_10_ = 100.8 Gy) for tumors located adjacent to major bronchus, esophagus, spinal cord, or great vessels, using 6 MV noncoplanar and nonopposing beams. At least eight beams were used. In response to the results of JCOG 07-02 trial [14] the prescription dose for peripheral lesions was raised up to 50 Gy in four fractions (BED_10_ = 112.5 Gy) from October 2010 and additionally up to 55 Gy in four fractions (BED_10_ = 130.6 Gy) from June 2013 to cover 95% of the planning target volume (PTV). Dose escalation scheme in JCOG0702 phase I study of SBRT for cases who were inoperable or refused surgery with clinical T2N0M0 nonsmall cell carcinomas was 50 Gy for D95 in level 3 and 55 Gy for D95 in level 4 for tumor volumes less than 100 cc. The results of that study are not yet available [14].

Conditions for recently reported prospective trials of SBRT for stage I NSCLC were as follows: irradiated dose was 20 Gy × 3 for T1 and 22 Gy × 3 for T2 in a phase II study (*n* = 70) by Fakiris et al. [15]; 15 Gy × 3 to 67% dose line of PTV in phase II (*n* = 57) by Baumann et al. [16]; 15 Gy × 3 (45 Gy) and 7.5 Gy × 8 (60 Gy) in phase II (*n* = 31) by Koto et al. [17]; 15 Gy × 3 in phase II (*n* = 62) by Ricardi et al. [18]; and 18 Gy × 3 in RTOG phase II (*n* = 55) by Timmerman et al. [19]. The 3-year local control of each report was 88.1% [15], 92% [16], 77.9% for T1 and 40% for T2 [17], 87.8% [18], and 97.6% [19].

The Radiation Therapy Oncology Group (RTOG) trial 0236 adopted a prescription dose of 60 Gy delivered in three fractions (BED_10_ = 180 Gy) to cover 95% of the PTV [20]. Our radiation dose is still lower than in the reports from Europe [7] and the United States [8, 20].

### 3.3. Definition of Target Volume in Our Institution

#### 3.3.1. Abdominal Compression

The patient was positioned in a supine position on a custom bed. A body cast was made to broadly cover the chest to the abdomen during shallow respiration and attached rigidly to the sidewall of the base plate.

**Figure 1:**
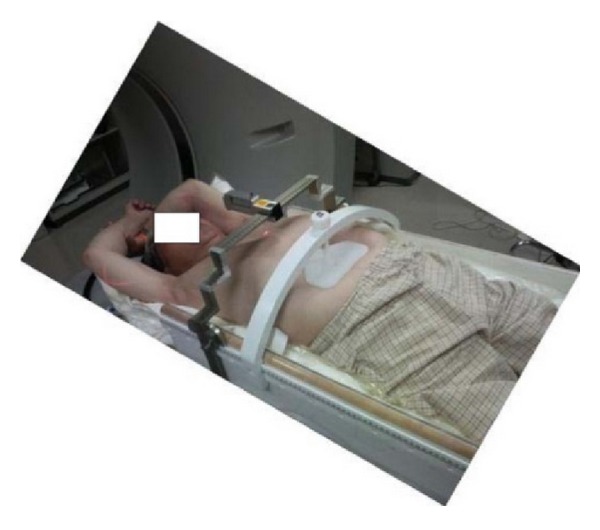
Body frame and abdominal pressure board.

A stereotactic body cast system was used with a custom bed and low temperature thermoplastic material RAYCAST (ORFIT Industries, Wijnegem, Belgium), at the University of Tokyo Hospital (Figure 1).

#### 3.3.2. ITV with Slow CT Scan

The CT images already included the internal motion because long scan time (four seconds) CT under free breathing (what is called, “slow” CT scan) was used [21, 22].

In our institution, from March 2003 to September 2010, CT images were acquired with 1 mm thick slices around the tumor and 5 mm slices elsewhere using the “long scan-time” technique, which visualized a major part of the trajectory of tumor movement by scanning each slice for a long time [23]. Slow CT scan was performed for 4 seconds with abdominal compression. These data were then sent to a treatment planning system (Pinnacle^3^ v7.4i; Phillips, Andover, MA, USA). The internal target volume (ITV) was delineated using the lung window, and PTVs were created by adding 5 mm margins to the ITVs in all directions.

The disadvantage of slow CT when compared with 4DCT may be that lung tumors with small motion cannot be accurately contoured on images, while, according to Nakamura et al. [24], the size difference between target volumes by slow CT and by 4D CT was not statistically significant.

#### 3.3.3. GTV and ITV with 4D CT

One improvement is that the four-dimensional (4D) CT for planning consists of 10 motion states, and gross tumor volume (GTV) was delineated in each respiratory phase from July 2010. The GTV was delineated using the lung window (window, 1600 HU; level, −300 HU), on the 10 respiratory phase CT datasets from 4D CT respiratory sorting. These 10 GTVs are fused to form the ITV and then a uniform 5 mm margin is added to create the PTV. Another improvement is similar to image-guided radiation therapy (IGRT), wherein a manual 4D registration for each fraction was performed to align the ITV contours with the tumor target presented in the “pre-4D” CBCT images which were taken just before irradiation for patient setup [25–27].

A large bore 16-multislice CT scanner of Aquillion LB (Toshiba, Japan), an Anzai belt (Anzai Medical, Japan), and a body fixation device of BodyFix (Elekta, Germany) were employed to obtain 10-phase respiratory-correlated CT data for a lung patient under constrained breathing conditions induced by an abdominal compression plate. A PTV was defined by adding a 5 mm margin to an internal target volume created from 10 GTVs, each of which was delineated on each phase of the 10-phase planning CT data. With regard to 4D CT construction protocol, the data acquisition time was 90–98 seconds, depending on the patient's size for taking whole lung 4D CT, and the slice thickness was 2 mm. CT image set for peak exhalation was used for treatment planning and for calculation of monitor units. Whole lung 4D CT was taken instead of only around the tumor.

#### 3.3.4. PTV-Leaf Margin

In Japan, in our institution as well as in others, a margin of approximately 5 mm between leaf and PTV is added in order to increase the homogeneity of radiation dose distribution within PTV. In JCOG 0403 [11, 12], the homogeneity index (HI) was set not to exceed the value of 1.6. The definition of our HI was maximum dose per minimum dose within PTV. This may increase in toxicities because of a larger radiation field. Because the higher radiation dose volume within PTV is smaller, this may decrease local control rate. In the near future, the idea that it is more important to guarantee the D95 or minimum dose within PTV than to hold down the HI will also be accepted in Japan.

### 3.4. Heterogeneity Correction in Our Institution

Since the lung is the most inhomogeneous site in the human body, it is very important for SBRT planning to take into account differences in tissue density in the dose computation and to accurately consider the secondary electron transport. Therefore, the use of advanced heterogeneous intensity-modulated radiation therapy correction and different types of algorithms has been recommended to calculate dose distribution accurately [28, 29].

Among various algorithms in commercial treatment planning systems, it is acknowledged that CCC can accurately predict the dose distribution [30]. In our institution, this CCC method as a heterogeneity correction has been adopted.

It is well known that the calculated target dose tends to be lower with CCC than with Clarkson. Generally, this implies that pencil beam-like algorithms such as in the Clarkson method tend to give the wrong impression that a good PTV coverage has been achieved when in reality this is not the case. The reason for this is lateral electron scattering, which is neglected by Clarkson [31]. Therefore, simple algorithms such as in Clarkson especially overestimate the dose in the interface between the target and lung tissue [32]. Our finding that the actual practice of relying solely on a Clarkson algorithm may be inappropriate for SRT planning in comparison with CCC and superposition (SP) has agreed with a previous study [33, 34]. From our study [35], an isocentric clinical dose calculated with the Clarkson algorithm is equivalent to approximately 1.2 times the PTV95 dose with CCC as a result of comparing dose distributions using 6 MV noncoplanar and nonopposing static beams (eight ports) with Pinnacle^3^ treatment planning system. The gantry and couch angles of the eight beams were 180° + 0°, 260° + 0°, 340° + 0°, 30° + 40°, 35° + 320°, 320° + 320°, 30° + 90°, and 330° + 90°, respectively [35].

To confirm safety and efficacy, SRT for lung cancer was under evaluation in multi-institutional clinical trials. For example, the JCOG conducted a phase II study 0403 of SRT in operable and medically inoperable patients with pathologically proven T1N0M0 NSCLC to evaluate efficacy and safety. Patient accrual for operable cases and their 3-year followup was completed in February 2010 [36]. Moreover, JCOG 0702, a phase I dose escalation study of SRT in patients medically inoperable or unfit for surgery with pathologically proven T2N0M0 NSCLC, was started to determine the recommended dose. In this context, in JCOG 0403, the prescribed dose was 48 Gy at the isocenter in 4 fractions and heterogeneity corrected doses by pencil beam convolution (PBC) algorithms were used since PBC could commonly be used in almost all clinical practices at that time. However, at the present time it is well known that PBC has shortcomings when it comes to severe inhomogeneities [37, 38]. As for lung cancer treatments, the actual dose was lower than expected. In JCOG 0702, therefore, the prescription was changed and the planning objective was for 95% of the PTV to be covered by the same isodose (i.e., 50 Gy) with SP or other newer algorithm than Clarkson.

In our institution, all plans with the exclusion of cases enrolled in JCOG 0403 were calculated with CCC algorithm using Pinnacle^3^ treatment planning system (TPS). It is useful to perform independent absorbed-dose calculations with the Monte Carlo (MC) algorithm in commissioning intensity-modulated radiation therapy (IMRT) [35, 39].

### 3.5. Position Adjustment Using 4D CBCT Immediately before SBRT

**Figure 2:**
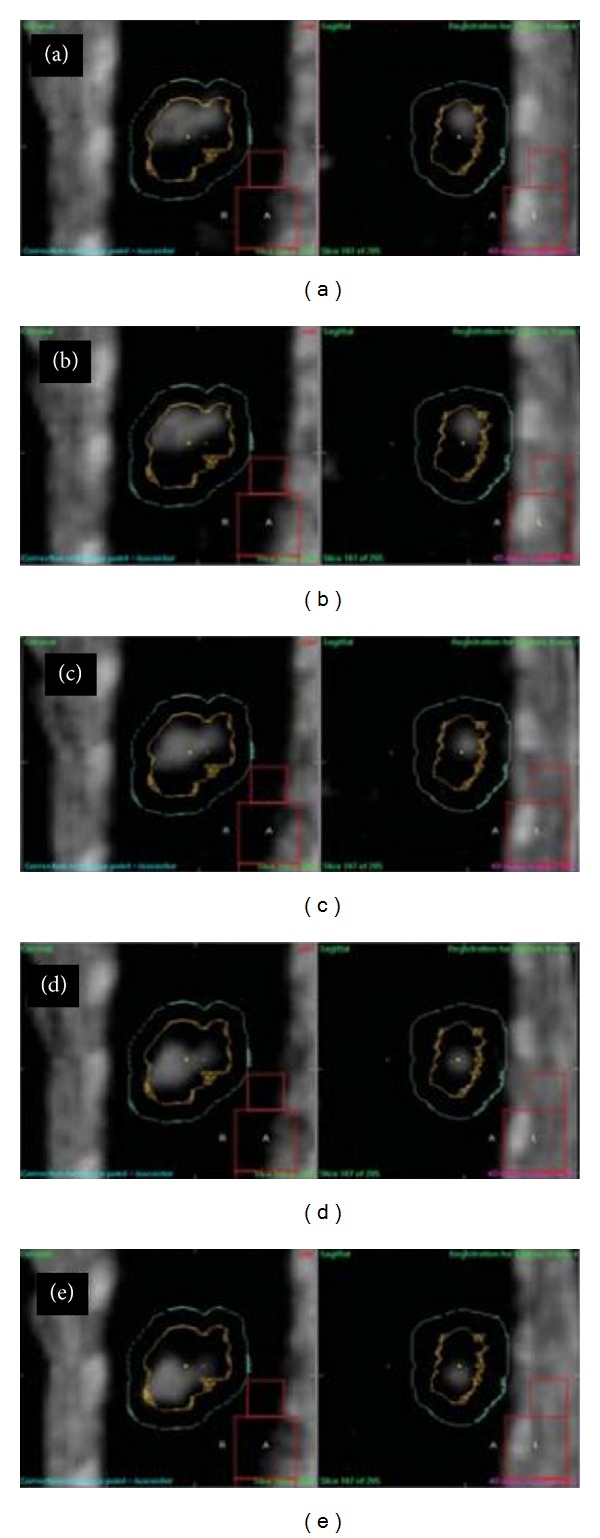
4D CBCT images on the first day overlaid with PTV (in sky blue) and ITV (in yellow) contours after lung tumor registration for five consecutive respiratory phases covering half a breathing cycle, where the tumor moves from cranial to caudal direction during the half cycle [9].

Three-dimensional (3D) “volumetric” imaging using CT mounted on the LINAC represents the latest development in the IGRT armamentaria [40]. Cone-beam CT (CBCT) imaging involves multiple kilovolt (kV) radiographs acquired by a large flat-panel detector [41–43]. The 4D CBCT was also extended by sorting kV radiograph images from the patient's respiratory signals before reconstruction [44, 45]. With the 3D or 4D information obtained just prior to treatment, the patient location can be corrected remotely by controlling the treatment couch, and the treatment can be quickly started. We showed 4D CBCT images overlaid with PTV and ITV contours after lung tumor registration for five consecutive respiratory phases covering half a breathing cycle in Figure 2.

We have developed an alternative respiratory correlated procedure for CBCT and evaluated its performance. This respiratory correlated CBCT procedure consists of retrospective sorting in projection space, yielding subsets of projections each corresponding to a certain breathing phase. Subsequently, these subsets are reconstructed into a 4D CBCT dataset. The breathing signal, required for respiratory correlation, is directly extracted from the 2D projection data, removing the need for an additional respiratory monitor system. Motion artifacts, clearly present in the 3D CBCT datasets, are substantially reduced in the 4D datasets, even in the presence of breathing irregularities, so that the shape of the moving structures can be identified more accurately. Moreover, the 4D CBCT dataset provides information on the 3D trajectory of the moving structures, absent in the 3D data. Considerable breathing irregularities, however, substantially reduce image quality. With respiratory correlated CBCT on a linear accelerator, the mean position, trajectory, and shape of a moving tumor can be verified just prior to treatment. Such verification reduces respiration induced geometrical uncertainties, enabling safe delivery of 4D radiotherapy such as gated radiotherapy with small margins.

**Figure 3:**
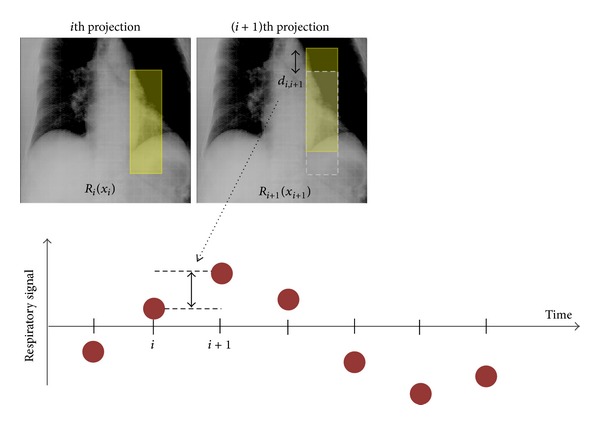
The outline of the image processing method in the image based phase recognition developed at the University of Tokyo Hospital.

4D CBCT images are reconstructed by classifying acquired projection images to respiratory phases divided by several bins. In this process, the knowledge of respiratory phases during projection imaging plays a key role.  Figure 3 shows the outline of the image processing method in the image based phase recognition developed at the University of Tokyo Hospital [46]. This method implements normal cross-correlation (NCC) between adjacent projections in a limited area, which is shifted along with the craniocaudal axis on the next projection image in searching for the maximum value of NCC with the segments on previous projection images. In general, a signal produced by an image-based phase recognition method includes a low periodic noise caused by the gantry rotation. This low periodic component can be removed by employing a high-pass (or band pass) filter.

**Figure 4:**
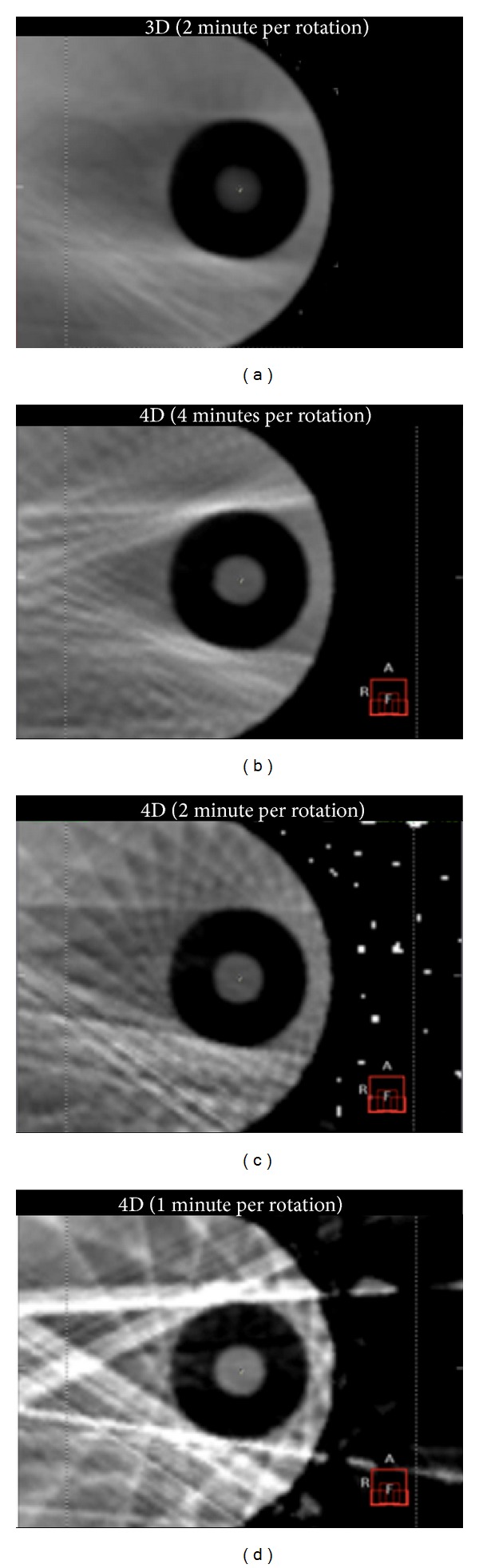
CBCT images (axial view) for a moving phantom (QUASAR; Modus Medical Devices, Inc.): (a) 3D (2 minutes per rotation), (b) 4D (4 minutes per rotation), (c) 4D (2 minutes per rotation), and (d) 4D (1 minute per rotation) images.

The space-time information of a tumor location from the clear images of 4D CBCT would play an important role in the delivery of precise radiation therapy. However, it should be noted that the slower gantry speed in 4D CBCT imaging could add a significant radiation dose to the patient. Therefore, it would be desirable to optimize radiation parameters to reduce the imaging dose as low as reasonably achievable. The mA per frame and ms per frame are 20 mA/frame and 40 ms/frame, which are used clinically in the University of Tokyo Hospital (Figure 4). With those parameters, the CT dose index (CTDI) volume is approximately 12 mGy for 4D CBCT imaging with 4 minutes per rotation, measured with a 15 cm length CTDI phantom.

### 3.6. VMAT for SBRT

As discussed in the literature, there are some challenges in using IMRT with regard to a moving target and other organs [47, 48]. Clearly, if a movement occurs between delivery of any of the IMRT fields, the dose may not add up to the desired total dose as planned. If there is organ movement during the delivery of a single IMRT field, the delivered intensity and dose map can also be very different from the planned one. This is known as the “interplay effect.” To avoid the interplay effect, the constraint on MLC motion of 0.1 cm/degree was applied in the VMAT inverse plan so that MLC had little chance to hide the PTV and carried out in accordance with the protocol in The University of Tokyo Hospital. The interplay effect with this constraint was negligibly small—which was determined by 4D dose reconstruction analysis using in-treatment 4D CBCT and LINAC log data [49].

The report from Mayo Clinic to validate the use of 50 Gy SBRT in 5 fractions using IMRT 7 noncoplanar beams for 26 patients with medically inoperable Stage I lung cancer was published [50]. The use of IMRT during SBRT has not been without issues for some authors, with questions on the feasibility of IMRT delivery within small fields typical of SBRT [51] and concerns that organ motion could negate the benefits of the IMRT [52]. Recently, it was reported that VMAT, which is a novel rotational technique and an extension of IMRT, is applicable for SBRT for lung tumors [53–55]. This technique achieves treatment plan qualities comparable to the noncoplanar IMRT technique and dramatically decreases the total treatment time for each fraction [56].

**Figure 5:**
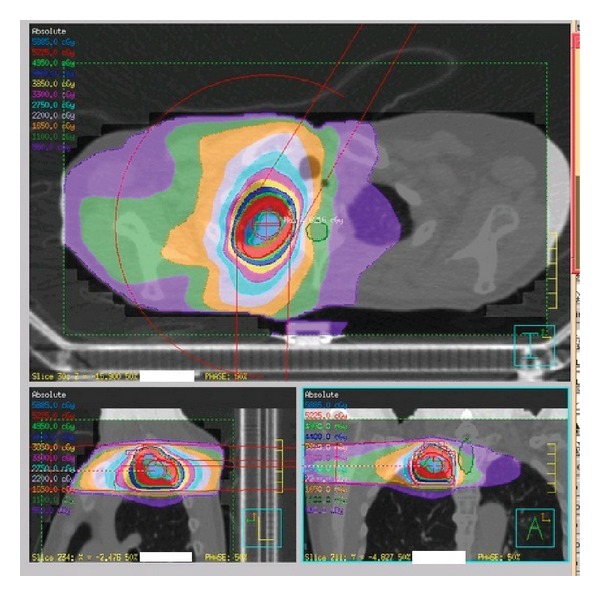
Radiation dose distribution of VMAT-SBRT.

We have performed VMAT-SBRT for primary or metastatic lung cancer patients (Figure 5). From October 2010 to December 2013, 67 consecutive lung cancer patients received single-arc VMAT-SBRT using an Elekta-synergy system. All patients were treated with an abdominal compressor. Treatment was performed with a D95 prescription of 50 Gy (43 cases) or 55 Gy (12 cases) in 4 fractions for peripheral tumors or 56 Gy in 7 fractions (12 cases) for central tumors.

The single-arc VMAT-SBRT with 6 MV was created by SmartArc (Pinnacle^3^; Philips). Dose constraints for normal organs at risk for complications were the ipsilateral lung volume receiving 20 Gy (V20) <10% and 5 Gy <25%; contralateral lung volume receiving 20 Gy (V20) <0% and 5 Gy <15%; spinal cord volumes receiving 15 Gy (V15) <0%; heart volumes receiving 30 Gy <0%; liver volume receiving 30 Gy <0%; and body receiving 50 Gy <0%. Dosimetric planning and plan analysis were performed in Pinnacle^3^. The CCC method in Pinnacle^3^ was used as the heterogeneous correction method for the lungs. All final calculations were performed with a grid size of 2.0 mm. Dose distributions were calculated using peak exhalation CT data.

The median followup was 267 days (range, 40–1162 days). Tissue diagnosis was performed in 41 patients (61%). There were T1 primary lung tumors in 42 patients (T1a in 28 patients and T1b in 14 patients), T2 in 6 patients, T3 (direct invasion to chest wall) in 3 patients, and metastatic lung tumors in 16 patients. The median mean lung dose was 6.87 Gy (range, 2.5–15 Gy). Six patients (9%) developed grade 2–5 radiation pneumonitis steroid administration needs. Actuarial local control rates for primary and metastatic lung cancers were 100% and 100% at 1 year, 92% and 75% at 2 years, and 92% and 75% at 3 years, respectively (*P* = 0.59). Overall survival rates for primary and metastatic lung cancers were 83% and 84% at 1 year, 76% and 53% at 2 years, and 46% and 20% at 3 years, respectively (*P* = 0.12). Use of VMAT-based delivery of SBRT in primary and in metastatic lung tumors demonstrated excellent local control and favorable survival. In order to improve these poor outcomes, some strategies such as dose escalation for metastatic lung cancers may have to be considered. Some metastatic lung tumors are known to be radioresistant such as those from colorectal cancers [57].

With respect to our clinical outcomes before employing VMAT, the control rate within the radiation field was 86.3% (101/117 cases) [13]. Out of the 117 cases, 74 patients had primary lung cancers and 43 patients had metastatic/recurrent cancers. The 117 cases were given SBRT from 2003 to 2009 in our institution. With respect to tumor control, our clinical outcomes were not improved by VAMT. About toxicities, since a shadow of interstitial pneumonitis on the CT image as well as biological markers (KL-6 and SP-D) before performing SBRT was used as an indicator for radiation pneumonitis after 2006, it is hard to evaluate whether the occurrence frequency of radiation pneumonitis after VMAT decreases.

### 3.7. Verifying Tumor Position within ITV Using In-Treatment 4D CBCT during VMAT-SBRT

**Figure 6:**
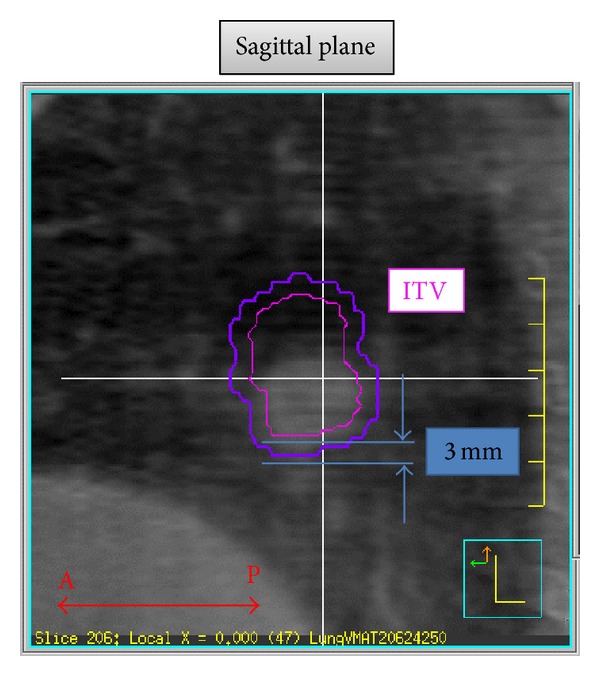
Example of the detection of sagittal displacement between the ITV and the actual tumor location, for which the ITV contoured on the planning CT was superimposed onto the in-treatment 4D CBCT image.

CBCT integrated into a radiation therapy system is a powerful tool in IGRT. The CBCT images acquired just prior to treatment enable us to localize the target accurately and to correct patient positioning. In addition, they have been used in planning adaptive radiation therapy during the course of the treatment [49, 58–61]. However, the CBCT images before treatment may be displaced from the actual location during treatment. Therefore, the ideal is to obtain the image volume in the state of delivered beams, and this is called “in-treatment CBCT” (Figure 6). In our previous paper, we reported that in-treatment CBCT can actually be acquired with rotational treatment such as VMAT [53], and displacement of the target can be evaluated using the volumetric images [62].

Setup error and tumor motion were evaluated during beam delivery by using 4D CBCT and the adequacy of the PTV margin was assessed for lung cancer patients undergoing VMAT for SBRT [56]. In the study, a total of 55 4D CBCT sets during VMAT-SBRT were successfully obtained and the amplitude of tumor motion was less than 10 mm in all directions. The average displacements between ITV and actual tumor location during treatment were 0.41 ± 0.93 mm, 0.15 ± 0.58 mm, and 0.60 ± 0.99 mm for the craniocaudal, left-right, and anteroposterior directions, respectively. The discrepancy in each phase did not exceed 5 mm in any direction.

The inter- and intrafractional respiratory motions of moving targets such as lung tumors and setup errors are significant concerns even for VMAT-SBRT. Inasmuch as the ITV setting accounts for respiratory motion and breathing patterns, the tumor motion may change between the simulation and treatment sessions. Therefore, the tumor position must be managed similarly during both simulation and treatment.

Monitoring and recording of the patient (or target) motion during treatment remain important and challenging topics for radiation therapy. Ideally the image volume is obtained in the state of delivered beams with gantry rotation using a technique called in-treatment CBCT. Recently, a system for performing in-treatment respiration-correlated CBCT, namely, 4D CBCT, was developed by using an image-based recognition technique of the respiration phase [46]. These in-treatment 4D CBCT images are most reliable for evaluating displacement during treatment. With this technique, the uncertainties of patient setup and moving targets can be clearly observed.

Our system could capture the tumor edge and provide a reasonable visualization of tumor location during treatment [9, 63]. Unlike pre-3D CBCT which was taken just before irradiation for patient setup, successful tracking of tumor location using in-treatment 4D CBCT could provide the answer regardless of the delivery of appropriate irradiation. In addition, ITV and PTV settings were evaluated in VMAT-SBRT with in-treatment 4D CBCT by comparing the tumor positions between the ITV (contouring based on 4D CT for treatment planning) and in-treatment 4D CBCT images simultaneously acquired during VMAT delivery.

Daily in-treatment 4D CBCT does not prolong treatment time. However, one of the problems of using in-treatment 4D CBCT is the additional exposure to kV X-ray irradiation. The total radiation exposure of in-treatment 4D CBCT scan was estimated to be as low as 30 mSv per day with our protocol, whereas the radiation exposure of pre-3D CBCT was approximately 15 mSv. These CBCT acquisitions delivered 4.5 cGy/fraction, which would result in an additional 18 cGy to a patient who received 50 Gy throughout the treatment period.

Although in-treatment 4D CBCT imaging could provide an accurate verification for clinical treatment, it is difficult to identify baseline shifts in the tumor position, which are manifested as smaller apparent breathing motion and larger apparent tumor size. This is because the present 4D CBCT technique does not provide real-time respiratory motion but instead presents an “averaged” one.

### 3.8. Shortening of Treatment Time Using Flattening-Filter-Free (FFF) Techniques

VMAT serves as a means for stereotactic hypofractionated treatment of lung tumors [64]. We proposed an efficient VMAT sequence by restricting leaf speeds of a multileaf collimator per gantry rotation angle to below 1 mm/degree, thereby reducing the dose delivery time down to 210 s for a D95 prescription dose of 50 Gy in four fractions [63]. We also suggested further reduction of the dose delivery time to below 100 s using FFF techniques [9].

We have proposed 4D digitally reconstructed radiography (DRR) for verifying a lung tumor position during VMAT [63]. During VMAT delivery, CBCT projection data were acquired by an onboard kilovoltage X-ray unit and a flat panel 2D detector. Four CBCT image sets with different respiratory phases were reconstructed using in-house software, where respiratory phases were extracted from the projection data.

We proposed a clinical workflow of stereotactic VMAT for a lung tumor from planning to tumor position verification using 4D planning CT and 4D CBCT [59]. The PTV contours were exported to a kilovoltage CBCT X-ray Volume Imaging (XVI) monitor equipped with a linear accelerator. Immediately before treatment, 10-phase 4D CBCT images were reconstructed leading to animated lung tumor imaging. Initial bone matching was performed between frame-averaged 4D planning CT and frame-averaged 4D CBCT datasets. Subsequently, the imported PTV contours and the animated moving tumor were simultaneously displayed on the XVI monitor, and a manual 4D registration was interactively performed on the monitor until the moving tumor was symmetrically positioned inside the PTV. A VMAT beam was delivered to the patient and during the delivery further 4D CBCT projection data were acquired to verify the tumor position. The entire process was repeated for each fraction.

Recently we evaluated a nonclinical FFF research configuration by adding a FFF to our LINAC system, a synergy with an integrated multileaf collimator (MLCi) (Elekta AB, Stockholm, Sweden) [10]. The FFF is made of stainless steel and has a constant thickness of 2 mm for 6 and 10 MV beams. Furthermore, we also tested a nonclinical onboard CBCT system, XVI 5.0 research version, which allows concurrent 4D CBCT imaging during VMAT delivery. It is anticipated that the reduced VMAT delivery time may degrade CBCT image quality due to a much lower number of projection images.

**Figure 7:**
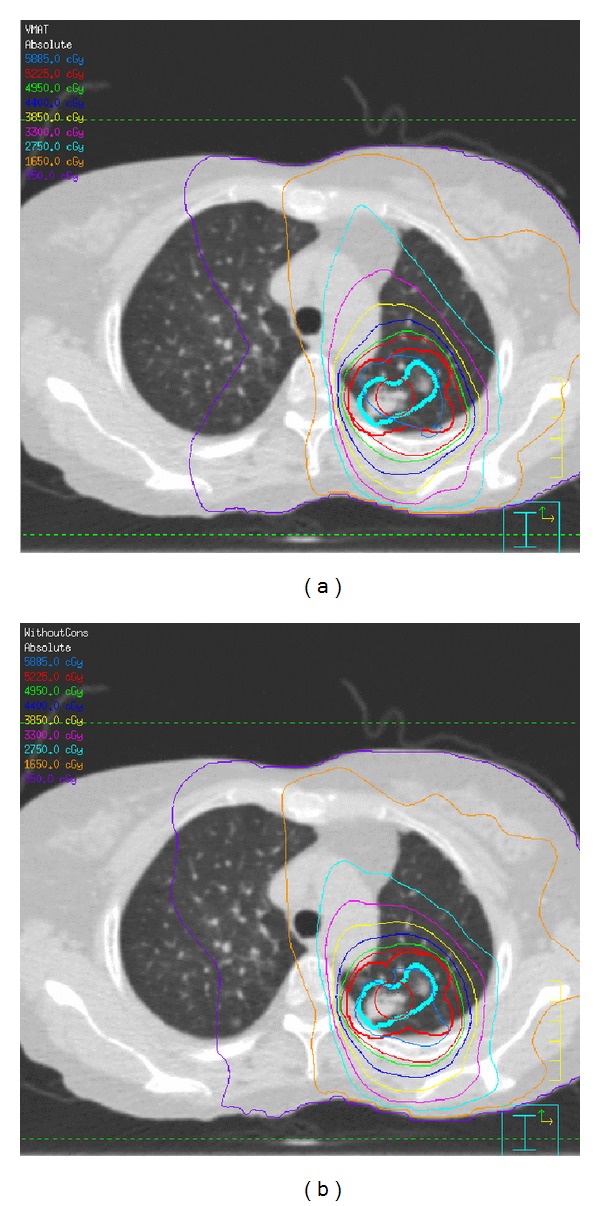
A comparison of calculated dose distributions for the VMAT plan with (a) FF and (b) FFF [10].

We have successfully shown an advantage of the FFF configuration in terms of dose delivery times for stereotactic lung VMAT [10]. A shorter delivery time ensures more accurate tumor positioning during treatment and possibly greater tumor control. It was also demonstrated that 4D CBCT-based tumor registration is feasible with FFF delivery (Figure 7).

### 3.9. Delivered 4D Dose Calculation for Lung-VMAT Patients Using In-Treatment CBCT and LINAC Log Data

**Figure 8:**
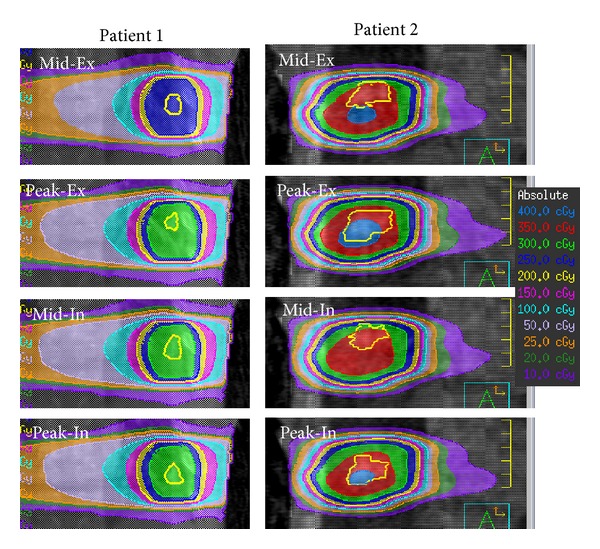
Dose distributions in each phase at the first fraction for 2 patients (coronal view). The yellow contour in each image indicates the gross tumor volume.

**Figure 9:**
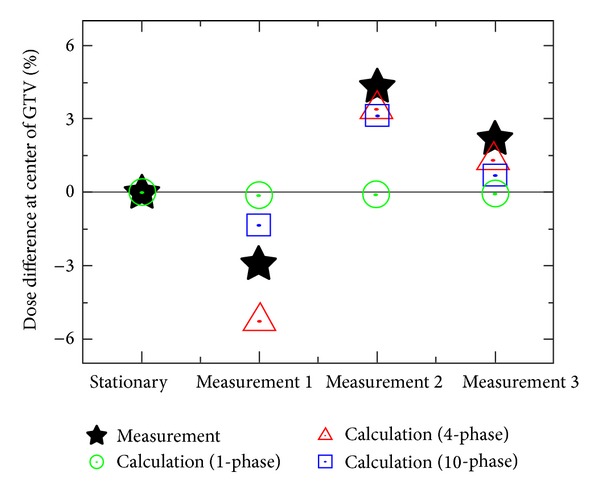
Relative dose difference at the center of target with and without motion using the QUASAR phantom. Here, the calculated dose was normalized at the measurement dose without motion (“Stationary” indicated in horizontal axis).

We developed a verification method for moving targets using 4D dose calculation based on the information acquired during treatment [65] (Figure 8). The beam shape, direction, and intensity were constructed from the LINAC log data, which was in excellent agreement with the EPID measurement for the MLC location. In the process of 4D dose calculation, sorting log data into breathing phase subsets was included. With corresponding log data, the dose calculation was performed on each phase of in-treatment 4D CBCT by means of the ROI mapping method. These 4D dose distributions demonstrated the delivered 4D dose distribution including interplay effect. The predicted dose value of the center of the target in a moving phantom agreed well with the measured dose (Figure 9).

### 3.10. Agility Multileaf Collimator

**Figure 10:**
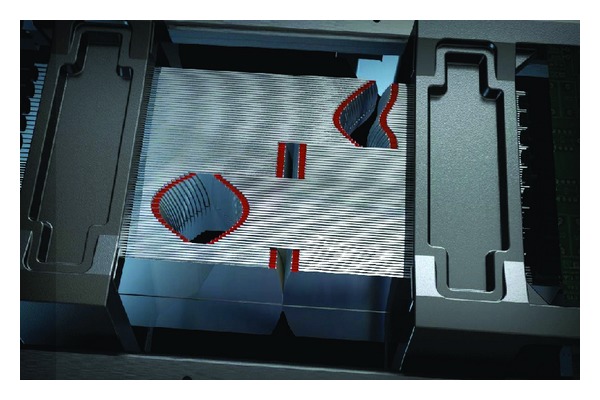
Agility multileaf collimator (http://www.elekta.co.jp/products/agility.html).

We use the latest Elekta MLC, Agility (Elekta AB, Stockholm, Sweden), for MLC tracking during VMAT. The Agility MLC has 160 leaves, with projected 5 mm leaf width at the isocenter, arranged in two banks of 80 leaves (Figure 10). Each bank of leaves is contained within a dynamic leaf guide (DLG), which can move with the MLC leaves. The maximum velocity of the individual MLC leaves is 35 mm/s, and for the dynamic leaf guide, 30 mm/s. Therefore the maximum possible velocity, if both the dynamic leaf guide and the MLC leaves are moving in the same direction, is 65 mm/s. There are two jaws that move perpendicular to the direction of MLC leaf travel, and these have a maximum velocity of 90 mm/s. These increased leaf and jaw velocities compared with previous Elekta MLC models, such as the MLCi with a maximum leaf speed of 20 mm/s, could confer an advantage for dynamic MLC tracking during VMAT delivery [66].

### 3.11. SBRT for Centrally Located Lung Tumor

SBRT for centrally located lung tumors including hilum lymph node metastasis remains a challenge because the central thoracic structures are considered to include organs at risk. For normal tissues, the use of a high dose per fraction rather than a conventional fractionated dose can increase the risk of late complications if the same volume were irradiated [67]. To date, only a few studies have reported on the safety and efficacy of treating centrally located lung tumors with SBRT [68–70]. In a group of 63 patients with centrally located lung tumors, Haasbeek et al. reported that a total of seven patients developed grade 3 acute or late toxicity after undergoing eight fractions of 7.5 Gy [71]. Rowe et al. also reported the results of SBRT for 47 patients with centrally located tumors. In their series, a total of five patients (10.6%) experienced grade 3–5 pulmonary toxicity [72].

**Figure 11:**
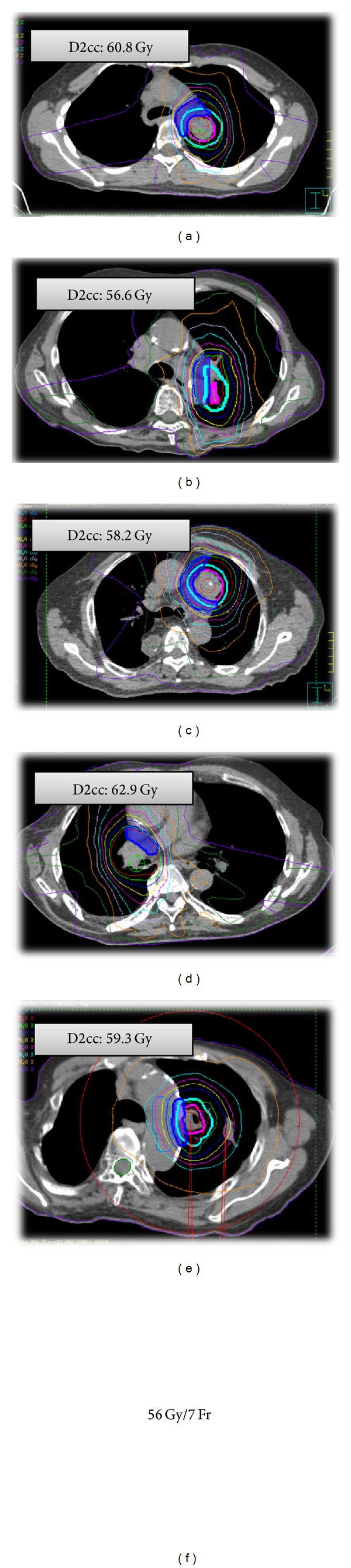
CT images showing dose distributions of the patients with top five cases of the minimum doses in the most irradiated 2 cc of the mediastinal structures (D2cc). Cyan, internal target volume (ITV); pink, planning target volume (PTV).

A centrally located tumor was defined as being within 2 cm of the bronchial tree, major vessels, esophagus, heart, trachea, pericardium, brachial plexus, or vertebral body. The planned dose at the PTV95% was 56 Gy in seven fractions (BED_10_ = 100.8 Gy) (Figure 11). Our retrospective study [73] demonstrated that SBRT for 45 centrally located lung tumors resulted in excellent local tumor control. The 2-year LCR of 77.3% compared favorably with other rates reported in the literature. As for the 2-year OS, there was a significant difference between primary NSCLC (*n* = 32) and pulmonary metastasis (*n* = 13) (69.4% versus 46.9%, *P* = 0.04) after the median follow-up time of 21.2 months. Since new metastases occur frequently in patients with lung metastases, they might carry a poor prognosis after successful delivery of the first SBRT.

## 4. Conclusions

In our institution, the procedures described here have been explored in order to raise the local control rate and decrease adverse effects after SBRT.
